# Prevalence and gender - specific analysis of a systemic sclerosis cohort in Latvia

**DOI:** 10.1186/s13023-024-03355-y

**Published:** 2024-09-30

**Authors:** Kristine Ivanova, Olga Ribakova, Anna Mihailova, Evelina Mozeitovica, Anda Kadisa, Julija Zepa, Viktorija Kenina, Natalja Kurjane, Inita Bulina

**Affiliations:** 1 Department of Doctoral Studies, Rīga Stradinš University, Riga, Latvia; 2 Institute of Oncology and Molecular Genetics, Rīga Stradinš University, Riga, Latvia; 3 Department of Rheumatology, Pauls Stradiņš Clinical University Hospital, Riga, Latvia; 4 Department of Residency, Rīga Stradinš University, Riga, Latvia; 5 Department of Internal Diseases, Rīga Stradinš University, Riga, Latvia; 6 ORTO Klīnika, Riga, Latvia; 7 Faculty of Medicine, Rīga Stradinš University, Riga, Latvia; 8 Institute of Microbiology and Virology, Rīga Stradinš University, Riga, Latvia; 9 Riga East University Hospital Gaiļezers, Riga, Latvia; 10 Department of Biology and Microbiology, Rīga Stradinš University, Riga, Latvia; 11 Department of Neurology, Pauls Stradiņš Clinical University Hospital, Riga, Latvia; 12 European Reference Network for Rare Neuromuscular Diseases, Paris, France; 13 Clinic of Medical Genetics and Prenatal Diagnostics, Children’s Clinical University Hospital, Riga, Latvia; 14 Centre for Clinical Immunology and Allergy, Pauls Stradiņš Clinical University Hospital, Riga, Latvia; 15 European Reference Network on Rare and Complex Connective Tissue and Musculoskeletal Diseases, Pisa, Italy

**Keywords:** Systemic sclerosis, Demography, Latvia, Prevalence of systemic sclerosis, Rhaynauds’s phenomen, Modified Rodnan skin score, Antinuclear antibodies

## Abstract

**Background:**

Systemic sclerosis (SSc) is considered by many to be one of the most severe autoimmune rheumatic diseases with lower prevalence observed in Northern Europe. No previous studies on the prevalence of SSc in Latvia have been conducted and the aim was to study the demographic and clinical data of patients with SSc in northeastern Europe country.

**Methods:**

This study was conducted in two main Latvian hospitals for adults and includes patients with SSc who were consulted between 2016 and 2021.

**Results:**

During the study period, 159 patients with SSc were consulted. The point prevalence on 1 January 2021 was 84.0 per million. Female to male ratio was 4.67:1, and highest gender ratio was observed in the age group 70–79-year (6.75:1). Antinuclear antibodies were present in 82.58% of patients, without gender difference. Centromere pattern was more frequently observed in females (40.19% vs. 19.04%), in contrast to speckled pattern (50.98% vs. 57.14%). At disease onset females tended to be younger (46.51 ± 13.52) than males (50.5 ± 16.64). Males had more diffuse cutaneous subtype, interstitial lung disease, pulmonary hypertension and esophageal dysmotility. More than half of patients received treatment with glucocorticoids at any point of the disease (68.31%), without gender difference.

**Conclusions:**

Systemic sclerosis is less common in Latvia than in other countries and regions. Due to its location, the data from Latvia are consistent with a north-south gradient in Europe. Gender ratio differences persisted in older age groups as well. Antinuclear antibodies presence did not differ between genders, but in female’s centromere pattern was much more likely to be present. Males had more severe disease course, but in both genders more than half of patients received treatment with GCs at any point of the disease.

**Supplementary Information:**

The online version contains supplementary material available at 10.1186/s13023-024-03355-y.

## Introduction

The term ‘scleroderma’ has been used since the mid-19th century but the first records date back to 1753, when Carlo Curzio described a 17-year-old girl with marked hardening of the skin all over her body [1]. Since 1980, scleroderma has been defined as a spectrum of diseases that consist of localized scleroderma and systemic sclerosis (SSc) [2]. Of the two types, localized scleroderma is more frequent with an incidence of 2.7 cases per 100,000, is not usually associated with severe systemic symptoms or Raynaud’s phenomenon and often is self-limited with a good prognosis [3].

On the other hand, SSc is considered by many to be one of the most severe autoimmune rheumatic diseases [4]. To verify the truth of this statement, accurate epidemiological data are needed. However, incidence and prevalence vary greatly between different studies, explained mainly by random sampling errors and differences between case definitions and capture methods. Also, SSc is a chronic disease, so its prevalence is influenced by incidence and mortality rates [5].

The average prevalence of SSc worldwide is estimated as 1 in 6500 adults. Lower prevalence (below 150 cases per million) and incidence (below 10 cases per million per year) are observed in Northern Europe and Japan, whereas higher incidence rates are observed in Southern Europe, North America and Australia [6–8].

As with other rheumatic diseases, the incidence of SSc varies according to gender. It is observed to be higher in females (female: male ratio of 3:1) [9], with a higher gender ratio for younger patients (7:1) but lower after the age of 50 years (2:1) [10]. The estimated average age of onset is 50 years. However, after the age of 75 years, the development of the disease is rarely seen [11, 12].

Gender differences explored in systemic connective tissue diseases may play an important role in early diagnosis and more accurate prognosis. There is already established higher premature death risk in males with SSc, and more severe expression of the disease, comparing with females with SSc [13].

No previous studies on the prevalence of SSc in Latvia have been conducted. Latvia is a country in northeastern Europe with a small population, a homogeneous race and two predominant ethnicities. Considering the above-mentioned national population characteristics, by studying the demographic and clinical data of SSc patients we could obtain previously unexplored data that will provide additional information on the characteristics of SSc in northeastern Europe.

## Materials and methods

### Subjects

This study was conducted in two leading Latvian hospitals, which are the only university hospitals in Latvia for adults.

Patients diagnosed with SSc who met the ACR/EULAR 2013 classification criteria and were consulted by rheumatologists between January 2016 and December 2021 were included [14].

For patient selection we used hospitals databases, where patients with diagnostic codes M34.0–M34.9 were selected according to the 10th revised version of the International Classification of Diseases (ICD-10), which has been used in all Latvian hospitals. Patients with connective tissue diseases other than SSc and patients with localized scleroderma were excluded. The study was approved by the Riga Stradins University medical ethics committee (Institutional Review Board reference no: 22 − 2/481/2021) and all participants provided written informed consent.

### Methods

To assess the presence and pattern of antinuclear antibodies (ANA), previously detected immunological tests were analysed. Analyses were carried out in two laboratories across both clinics. However, ANA were detected using Hep-2 cells in one laboratory at Paul Stradins Clinical University Hospital for all patients in this study.

Patients who agreed to participate in the study were evaluated by one rheumatologist and surveyed and clinically assessed according to the European Scleroderma Trials and Research (EUSTAR)-accepted domains. The domains created by EUSTAR in 2015 include the collection of demographic data, patient complaints, the evaluation of skin conditions according to the modified Rodnan skin score (mRSS) [15]. Interstitial lung disease (ILD) and pulmonary hypertension (PH) were determined after previous investigations including lung computed tomography (CT), transthoracic echocardiography (ECHO), and right heart catheterisation (RHC). Esophageal dysmotility was assessed by patient complaints and previous upper gastrointestinal series.

The age at disease onset was defined as the time of onset of the first non- Raynaud’s SSc symptom.

The classification of patients according to subtypes of SSc (diffuse, limited, sine-scleroderma) was not determined during this study, but took into account information provided in previous database.

### Statistical analysis

Statistical analysis was performed using SPSS 22.0 software (SPSS Inc., Chicago, IL, USA). Data normality was assessed using histograms and the Kolmogorov-Smirnov test. For comparison between the groups, the Kruskal-Wallis H test, Spearman’s rank-order correlation and Fisher’s exact tests were used; P values of < 0.05 were considered to be significant.

## Results

### Prevalence

Between January 2016 and December 2021, 159 patients with SSc were consulted in Latvia’s university hospitals. Of the 159 patients, the majority were females (82%) and only 18% were males. The mean patient age was 62.53 ± 12.11 years, with females slightly older (63.12 ± 11.54 years) than the males (59.75 ± 14.37 years).

On 1 January 2021 the population of Latvia was 1,893,223 and the point prevalence was 84.0 (95% CI = 71.9–98.1) per million (Table 1). The prevalence ratio was higher for females − 128.7 (95% CI = 108.5–152.7) than for males − 32.0 (95% CI = 22.1–46.2). When adjusted to the European standard population the total prevalence was 62.8 (95% CI = 57.8–67.7) and when adjusted to the WHO world standard population it was 49.9 (95% CI = 45.9–53.8).

**Table 1:** Age- and gender-specific prevalence rates (per million) of systemic sclerosis in Latvia, 1 January 2021

	Mean annual population			Number of cases			Prevalence rate (95% CI)			^a^*P* value
Age group (years)	Males	Females	Total	Males	Females	Total	Males	Females	Total	
20–29	98,147	91,079	189,226	1	2	3	10.2 (1.8–57.7)	22.0 (6.0-80.1)	15.9 (5.4–46.6)	0.038
30–39	138,148	130,603	268,751	3	5	8	21.7 (7.4–63.9)	38.3 (16.4–89.6)	29.8 (15.1–58.7)	0.032
40–49	124,588	128,280	252,868	2	5	7	16.1 (4.4–58.5)	39.0 (16.6–91.2)	27.7 (13.4–57.1)	0.002
50–59	122,174	139,441	261,615	5	30	35	40.9 (17.5–95.8)	215.1 (150.7-307.1)	133.8 (96.2–186.0)	< 0.001
60–69	103,142	141,844	244,986	11	54	65	106.6 (59.6–191.0)	380.7 (291.8-496.6)	265.3 (208.2-338.1)	< 0.001
70–79	57,721	111,032	168,753	4	27	31	69.3 (26.9-178.2)	243.2 (167.1-353.8)	183.7 (129.4-260.7)	< 0.001
80–89	25,710	72,230	97,940	2	8	10	77.8 (21.3-283.6)	110.8 (56.1-218.6)	102.1 (55.5–188.0)	0.016
Total	875,225	1,017,998	1,893,223	28	131	159	32.0 (22.1–46.2)	128.7 (108.5-152.7)	84.0 (71.9–98.1)	< 0.001
Adjusted to European standard population							26.7 (21.6–31.7)	90.5 (82.6–98.4)	62.8 (57.8–67.7)	< 0.001
Adjusted to WHO world standard population							21.8 (17.7–26.0)	71.7 (65.4–77.9)	49.9 (45.9–53.8)	< 0.001
	Males	Females	Total	Males	Females	Total	Males	Females	Total	

aP value for statistical differences between the rates for males and females

**Figure 1:**
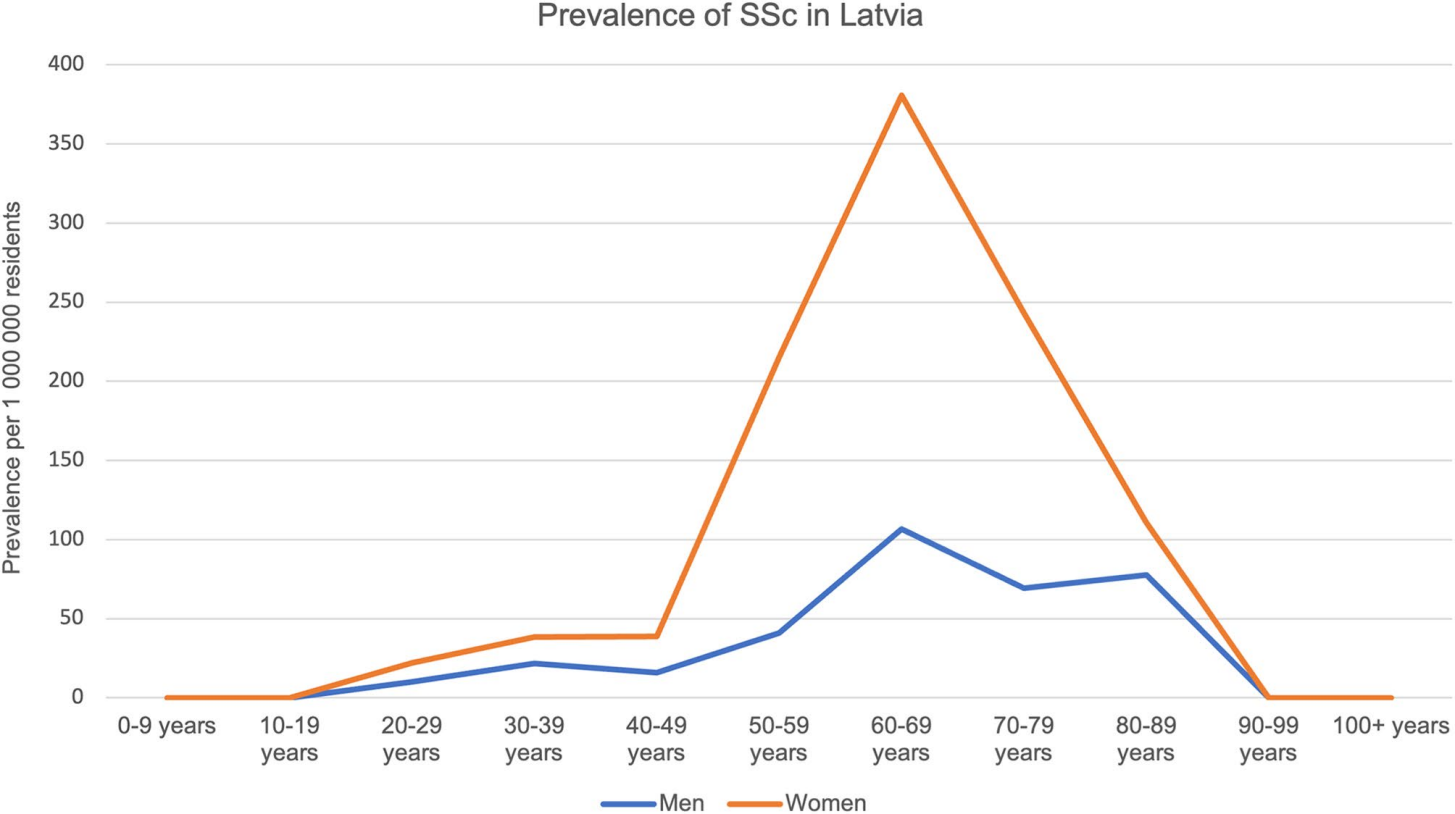
Fig. 1

The highest prevalence was found in the 60–69 age group (Fig. 1). In all groups, the rates for females were higher than for males. The difference was statistically significant in all age groups where patients were present.

### Antibody characteristics

The presence of ANA was evaluated and found in 82.58% of 155 patients (Table 2). The pattern was checked in 122 patients. Most patients had either speckled pattern (52.45%) or centromere pattern (36.88%). In speckled pattern group of 64 patients, 39 had anti-topoisomerase I. A few patients had homogeneous pattern (6.56%) or nucleolar pattern (5.74%). ANA were found almost equally in females (82.81%) and in males (81.41%), but a difference between genders was observed in ANA patterns. Centromere pattern was more frequently observed pattern in females than in males (40.19% vs. 19.04%), while speckled pattern was the most frequently observed pattern in both genders almost equally with slight male predominance (50.98% vs. 57.14%).

**Table 2:** Gender-specific antibody characteristics in patients with systemic sclerosis

		Males	Females	Total
Antinuclear antibodies	ANA positive (n) ANA pattern present (n)	22 21	106 102	128 123
	Centromere pattern (n (%))	4 (19.04%)	41 (40.19%)	45 (36.88%)
	Speckled pattern (n (%)) Anti-topoisomerase I] (n (%)	12 (57.14%) 7 (33.33%)	52 (50.98%) 32 (31.37%)	64 (52.03%) 25 (20.32%)
	Homogeneous pattern (n (%))	2 (9.52%)	6 (5.88%)	8 (6,50%)
	Nucleolar pattern (n (%))	3 (14.28%)	4 (3.92%)	7 (5.69%)

Clinical characteristics.

**Figure 2:**
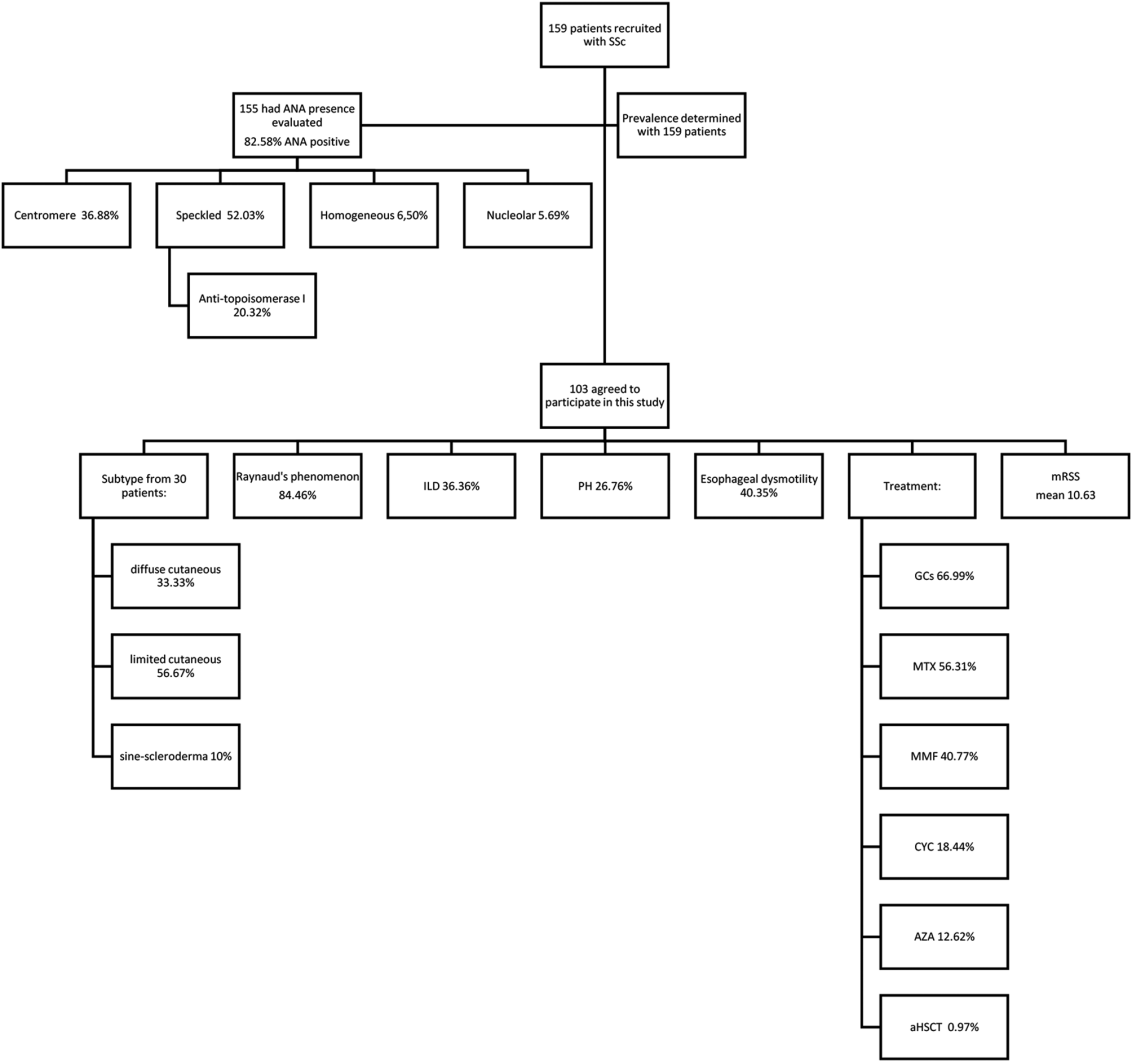
Fig. 2

Of the 159 patients selected, 103 agreed to participate in this study (Fig. 2), of whom 85 were females and 18 were males. All included patients were Caucasians.

Disease duration (from the first non- Raynaud’s symptom) ranges from 1 to 41 years. The mean age at disease onset was 47.21 (± 14.10) years and the females tended to be younger (46.51 ± 13.52) than the males (50.5 ± 16.64), (Table 3).

**Table 3:** Gender-specific clinical characteristics in patients with systemic sclerosis

		Males	Females	Total
Descriptive	Total count, *N*	18	85	103
	Minimum disease Duration, *years*	1	2	1
	Maximum disease Duration, *years*	21	41	41
	Mean (SD) disease duration, *years*	8.95 ± 6.33	15.14 ± 9.87	14.06 ± 9.62
	Minimum age of onset	14	5	5
	Maximum age of onset	80	74	80
	Mean (SD) age of onset	50.5 ± 16.64	46.51 ± 13.51	47.21 ± 14.10
Symptoms	Raynaud’s phenomenon, *N* (%)	16 (88.88%)	71 (83.52%)	87 (84.46%)
	modified Rodnan skin score, mean (SD)	10.36 ± 12.95	10.67 ± 8.78	10.63 ± 9.41
SSc types, *N* (from 30 patients)	Sine-scleroderma	0	3 (11.54%)	3 (10%)
	Limited	2 (50%)	15 (57.69%)	17 (56.67%)
	Diffuse	2 (50%)	8 (30.77%)	10 (33.33%)
Interstitial lung disease, *N* (from 99 patients)		7 (38.89%)	29 (35.80%)	36 (36.36%)
Pulmonary hypertension, *N* (from 71 patients)		4 (30.77%)	15 (25.42%)	19 (26.76%)
Esophageal dysmotility, *N* (from 57 patients)		5 (45.45%)	18 (39.13%)	23 (40.35%)
Treatment, *N* (%) (from 101 patients)	Glucocorticoids	12 (66.66%)	57 (67.05%)	69 (66.99%)
	Methotrexate	7 (38.88%)	51 (60.00%)	58 (56.31%)
	Mycophenolate mofetil	9 (50.00%)	33 (38.82%)	42 (40.77%)
	Cyclophosphamide	4 (22.22%)	15 (17.64%)	19 (18.44%)
	Azathioprine	2 (11.11%)	11 (12.94%)	13 (12.62%)
	Autologous hematopoietic stem cell transplantation	0	1 (1.17%)	1 (0.97%)

The majority (84.46%) of patients had Raynaud’s phenomenon. SSc types were only available for 30 patients, most common type was limited (56.67%), followed by diffuse (33.33%), and the least common was sine-scleroderma (10%). Sine-scleroderma was not observed in male patients, but the diffuse type was as common as the limited type.

The mRSS was evaluated in all 103 patients, with a mean score of 10.63, without difference between genders (10.67 in females and 10.36 in males).

99 patients had a CT scan available in the database. Of these, ILD was described in 36 patients (36,36%), slightly more often in males (35.80% in females and 38.89% in males). ECHO data was available for 71 patients, where PH was suspected and verified by RHC in 19 patients (26.76%), also more often in males (25.42% in females and 30.77% in males). Esophageal dysmotility was evaluated in 57 patients, and present in 23 patients (40.35%), less in females (39.13%) than males (45.45%).

More than half of the patients received treatment with glucocorticoids (GCs) at any point of the disease (66.99%). Methotrexate (MTX) was the next most frequently used immunosuppressant (56.31%), followed by mycophenolate mofetil (MMF) (40.77%). Almost a fifth of patients were treated with cyclophosphamide (CYC) (18.44%). One patient received autologous hematopoietic stem cell transplantation (aHSCT). While treatment with GCs was observed equally between genders, females were more often treated with MTX (60.88% vs. 38.88%) and males with MMF (38.82% vs. 50.00%). Out of 10 patients with diffuse type, 90% received GC therapy, less frequently with limited type (70.59%) and sine-scleroderma (66.67%). Of patients with evaluated SSc type and available lung CT, 8 of 14 (57.14%) received GC without known ILD or diffuse SSc type, (Table 4).

**Table 4:** Gender-specific glucocorticoid treatment in different systemic sclerosis types and interstitial lung disease

		Males	Females	Total
Glucocorticoids, *N* (%)	Sine-scleroderma	0	2 (66.67%)	2 (66.67%)
	Limited	1 (50%)	11 (73.33%)	12 (70.59%)
	Diffuse	1 (50%)	8 (100%)	9 (90%)
	Interstitial lung disease	6 (100%)	21 (100%)	27 (100%)
	No interstitial lung disease	6 (54.55%)	36 (69.23%)	40 (63.49%)

## Discussion

The significance of this study lies in the specificity of the country with a small population. There are only two hospitals for adults with an established team of rheumatologists, and we included both. Virtually all patients with suspected SSc in Latvia are referred to one of these hospitals, so we are effectively describing the general Latvian population by selecting and evaluating patients from these hospitals.

There are two main ethnic groups in Latvia – Latvians (62.1%) and Russians (26.9%) – along with other eastern Europeans (ca. 8%), Jews (0.3%) and Romani (0.3%) [16, 17]. The Latvian population mainly consists of Caucasians, as represented in our results with 100% Caucasians. In this study we did not distinguish patients’ ethnicity. Still our colleagues in Estonia (where two ethnic groups also predominate, Estonians and Russians) found that the prevalence of SSc, especially CREST syndrome, was higher among the Russians [18]. To clarify, the CREST terminology is no longer widely used, and the term used instead is limited cutaneous SSc, as mentioned in our study [19].

Only a few studies were conducted in Northern or Eastern Europe. One study carried out in southeast Norway found the prevalence of SSc to be compatible with other northern European countries, supporting the notion of a north–south gradient of SSc in Europe, with the lowest prevalence in Northern Europe [20]. Opposing results were presented from Sweden, where the prevalence was higher at 235 per million inhabitants [21].

In our study, the point prevalence was 84.0 (95% CI = 71.9–98.1) per million, which is lower than the results in review about 50 publications from Europe and North America, with reported prevalence of 70.2–333.9 and 135–443 per million in Europe and North America, respectively [22]. Although we cannot identify any specific reason for this, the relatively low prevalence is unlikely to be due to study shortages but rather to a possible shortage of rheumatologists in the country and the unavailability of consultations. This would be particularly true for patients with a limited subtype of the disease, without severe PH, who do not feel the need to visit their general practitioner.

We observed the highest prevalence in the 60–69 age group, that was not similar in other European countries. For example, in Sweden and Italy 70–79 age group had the highest prevalence [23, 24].

This study did not analyse incidence data for SSc. The main reason for this choice is missing data in hospital databases, and relying only on the medical history from patients can lead to very misleading data.

We report a higher mean age in this study for females than males: 63.12 versus 59.75 years. This was not seen in the Norway study, where the difference was minimal (56.7 versus 56.1 years) [20]. Also, the mean age of both genders was older than represented in other similar studies: 62.53 ± 12.11 years versus 50.8 ± 12.5 years in Italy [25] and 56.8 ± 12.2 years in Hungary [26].

A higher female predominance was seen in this study than is reported worldwide, with a female: male ratio of 4.67:1 compared to 3:1. However, it was similar to other European reports, where the ratio was estimated to be 3.8–11.5:1 [22], so the study of gender difference should probably be based on regional data rather than on global data linking very different regions together.

The highest gender ratio was observed in the 70–79-year age group (6.75:1), contradicting previous observations of a lower gender ratio after the age of 50 years (2:1) [10]. In younger patients we did find a lower gender ratio (2:1), but this again contradicted the worldwide data [10]. Of course, probabilities must be expressed with caution with the small number of patients. Still, in our study, we probably captured the characteristics of older men avoiding medical help in Latvia.

Most of patients evaluated were ANA positive, with anti-speckled and anti-centromere patterns present almost equally. The presence of ANA in patients with SSc is widely observed, with levels as high as 98% reported [27]. Three serum autoantibodies that are included in the 2013 classification criteria (anti-RNA polymerase III, anti-topoisomerase I and anti-centromere) account for over 70% of all single antibody specificities detected in previous studies [14, 27]. Unfortunately, at the time of study, it was not possible to detect anti-RNA polymerase III, but 84 patients (68.85%) from the 122 evaluated had either anti-centromere or anti-topoisomerase I. In recent data with knowledge of new antibodies associated with SSc, still highest prevalence stands for these two antibodies [28]. Contrary to our results, in the Norway study, there was significant anti-centromere predominance compared to anti-topoisomerase I (54.2% vs. 13.5%) [20]. Previously, many studies reported higher anti-centromere prevalence in Caucasians [29, 30]. In contrast, in a study from the USA, evaluating the prevalence of autoantibodies in a different race, only 17% of Caucasian patients had positive anti-centromere antibodies, with more (19%) having anti-RNA polymerase III [31]. We found that anti-centromere-positive patients were more likely to be females, whereas the difference was not as significant between anti-topoisomerase I positive males and females. In other studies, females were substantially more likely to have anti-centromere antibodies, whereas males more likely to have anti-topoisomerase I [27]. In our study, we present different data from the previous studies. With 100% Caucasian patients, there was no significant anti-centromere antibody predominance and there was a high prevalence of anti-topoisomerase antibodies. Although ANA positive patients were fewer than in majority of other studies, it could be higher with repeated examination dynamically [32].

Most of our patients presented with the first non- Raynaud’s SSc symptom in the fifth decade of life. Study from Sweden showed similar results (48 ± 4.1 years) [23]. However, disease onset is hard to determine and has not been defined similarly in other studies. The age at which the diagnosis was made is generally analysed and in data from Europe it varies in the range 33.5–59.8 years [22]. In our view, it is also essential to note patients’ observations of their first symptoms, allowing more reliable conclusions of differences between several populations. By contrast, if the focus remains on the time of diagnosis, we may mistakenly assess not the characteristics of the disease but the availability of specialists in different countries.

We report a slight age difference when comparing both genders at disease onset, with females being younger than males. Younger female age at onset is not uncommon, and other studies have presented similar findings. In a study from Greece, the age difference was markedly larger but, similarly, the females tended to be younger [33]. In Pittsburg, USA, the results were very similar to ours: 43.8 ± 14.0 years for females; 46.4 ± 13.7 years for males [27].

Although the number of males in the study was small, we observed a similar trend towards a more severe disease course, with more frequent development of ILD and PH, as in other studies [13, 34, 35]. As the main causes of SSc-related mortality, these data also explain the worse outcomes in males. However, there are no clear data on the difference in the incidence of esophageal dysmotility between genders. Historically, dysmotility was described as another close symptom to the limited subtype but we observed a higher frequency of dysmotility in males, although the limited form did not predominate as the most common subtype of disease in them [36, 37].

We found that more than half of patients (68.31%) received treatment with GCs at any point of the disease. Although this number is exceptionally high, the trend is not exclusive to our study. The German Network for Systemic Scleroderma data showed that 41,3% of all registered SSc patients were treated with GCs [38]. EUSTAR database provided very detailed data on GCs prescribing practices in SSc, with 34% off patients taking GCs at baseline of the study, but the use of GCs from disease onset was not included. There were no data from Latvia, but interestingly eastern Europe countries tended to prescribe GCs more [39]. In the most recent update of the EULAR recommendations for the treatment of SSc, the experts recognized that GCs, which are used in SSc, are part of the therapeutic strategy in the management of ILD, diffuse cutaneous disease or musculoskeletal involvement [40]. However, the evidence regarding their efficacy in SSc is limited [29]. In Latvia, the trend of GCs use was more pronounced in patients with diffuse cutaneous SSc, but it was also used in more than half of patients with limited cutaneous SSc and with sine scleroderma. The most difficult to explain the use of GCs was in 57% of patients who used them without diffuse skin involvement and ILD. Patients enrolled in the study were treated for up to several decades. We think this is also why the number of patients treated with GCs was so high. Previously, higher expectations were placed on GCs in the treatment of SSc. We did not analyse the use of GCs over time, but following further and more recent studies there is a high probability that the use of GCs will decrease in Latvia. It is more likely that, as knowledge of the role of immunosuppressive therapies in SSc develops, data will also show a positive trend towards a reduction in the use of GCs in Latvia.

We are aware of some limitations of the study. Due to the country specificity, most patients with suspected SSc are referred to the two university hospitals mentioned above, but we cannot exclude a number of patients who were nevertheless not included in this study. One of the reasons why a proportion of patients may not have been included in our study is the lack of capillaroscopy data at the time of diagnosis, which could be the reason why the SSc classification criteria were not met. Another shortcoming in meeting SSc classification criteria could be lack of evaluation of anti-RNA polymerase III.

## Conclusion

SSc is less common in Latvia than in other countries and regions. Due to its location, the data from Latvia are consistent with a north-south gradient in Europe. With its homogeneous racial pattern, Latvia is probably an even more pronounced model for the developing of SSc in northern countries. Female to male ratio was 4.67:1, and gender ratio differences persisted in older age groups with highest gender ratio observed in the age group 70–79-year. ANA presence did not differ between genders, but in females centromere pattern was much more likely to be present. Disease developed earlier in females, without significant difference in Raynaud’s presence or severity by mRSS. Males had more severe disease course, but in both genders more than half of patients received treatment with GCs at any point of the disease.

## Electronic supplementary material

Below is the link to the electronic supplementary material.

Supplementary Material 1

Supplementary Material 2

## Data Availability

The data that support the findings of this study are openly available in Synapse at 10.7303/syn56849053; syn56849053.
