# Supplementary material for: Prevalence and gender - specific analysis of a systemic sclerosis cohort in Latvia
Source: Orphanet J Rare Dis. 2024 Sep 30;19:361. doi: 10.1186/s13023-024-03355-y (PMC11443687; doi:10.1186/s13023-024-03355-y)
Supplement: Supplementary file 2 — Supplementary Material 2 [file 13023_2024_3355_MOESM2_ESM.docx]

Response to Reviewers

Reviewer #1: Major comments

- The title of the manuscript is misleading: the immunological and clinical characteristics are far from complete and I suggest to adapt the title to "prevalence and gender specific analysis of a SSc-cohort in Latvia"

Thank You very much for the suggestion to change the title of the manuscript, and we definitely agree. We changed the name of the manusctript: Prevalence and Gender-Specific Analysis of a Systemic Sclerosis Cohort in Latvia.

- The reasoning why the authors analyzed their data in a gender specific way is unclear.

Thank you very much for drawing attention to a missing explanation. Aware of the possibilities we can use to analyse a rare disease in a country with such a small population, we chose as one of the strengths of the study to also analyse gender differences in SSc, taking as an example another systemic connective tissue disease, systemic lupus erythematosus, where there are more data on sex differences to facilitate diagnostic choices for clinicians.

Thanks to the comment, the explanation in the introduction part is completed.

- Unfortunately, no details on disease characteristics were given. It would be of more importance to know what the prevalence of certain subtypes is: such as the limited cutaneous and diffuse cutaneous subset? Prevalence of PHT, ILD and digital ulcers?

Thank you very much for your professional persuasion, and for the absolute need to go into more detail about the disease.

It is clear that without this addition, the research carried out loses its relevance and usefulness.

Therefore, additional data on possible clinical manifestations such as pulmonary hypertension, interstitial lung disease, esophageal dysmotility were included.

Unfortunately, there were limited possibilities to categorise patients by type. We did not re-evaluate the types for fear of data inaccuracy due to the sometimes-long duration of illness and having already received immunosuppressive therapy. Pre-defined disease types were considered and analysed, following your very valuable guidance.

- The authors claim to have included patients based on 2013 EULAR SSc classification criteria, but did all patients received a nailfold capillaroscopy? Moreover, not all patients received a full immunoblot with antiRNApolymerase antibodies. Diagnosis could have been missed, which should be underlined in the discussion.

Thank you very much for this insight. You are absolutely right, capillaroscopy is an absolutely essential technique in the diagnosis and management of systemic sclerosis. In our country, unfortunately, the capillaroscopy method was not available at the time of the study. Also, the systemic sclerosis autoantibody panel, which included anti-RNA polymerase, was not available at the time from which the patients were recruited. You are absolutely right about the possible missed patients due to these shortcomings. Thanks to your comments, additional clarifying information was included in the discussion part.

 The study did not report on the presence of ILD, diffuse skin involvement and musculoskeletal involvement? Perhaps the patients having more severe disease are the one more frequently reaching out for medical care in Latvia? The inclusion of details on disease characteristics in this study would help to understand why epidemiological data differ with data from other countries. It would also help to understand why such a high rate of corticosteroid use is reported.

Thank you very much for your professional persuasion, and for the absolute need to go into more detail about the disease.

It is clear that without this addition, the research carried out loses its relevance and usefulness.

Therefore, additional data on possible clinical manifestations such as pulmonary hypertension, interstitial lung disease, esophageal dysmotility were included, also referring to GCs usage.

- The epidemiological data came from patients who consulted the two only adult academic hospitalis in Riga. Can the authors give some information about the access to health care in Latvia? Where the same rheumatologists seeing the patients in both centers?

Thank you very much for your interest in the availability of rheumatologists in Latvia.

Mostly, the two university clinics are staffed by different colleges, so it is likely that before the study, patients were consulted by several doctors from the same centre. However, rheumatologists who do not work at the university clinic refer patients with suspected systemic sclerosis to one of these two centres. Patient data were checked and compared, and in cases where a patient was consulted at both university clinics, no duplication of results was allowed.

Was then the same laboratory clinics involved for both centers and on what cell line has these been examined? Hep2000-cells?

Thank you for your questions regarding antibody detection. Antibodies were mainly detected in two laboratories across both clinics. However, ANA were detected using Hep2 cells in one laboratory at Paul Stradins Clinical University Hospital for all patients in this study.

- Were no immunoblots been used? Was the centromere pattern confirmed by an extractable antigen blot with CenpB?

Thank you for your questions regarding antibody detection again. The systemic sclerosis autoantibody panel, which included anti-RNA polymerase, was not available at the time the patients were recruited. Due to this limitation, it is possible that some patients were missed. Thanks to your comments, additional clarifying information has been included in the discussion section. Unfortunately, the centromere pattern was not confirmed by an extractable antigen blot with CenpB.

Minor comments:

- Can the authors describe what were the reported first "non-Raynaud's symptoms"?

Thank you for highlighting this serious issue. The symptoms associated with systemic sclerosis, excluding Raynaud's phenomenon, include those listed in the classification criteria as puffy fingers, sclerodactyly, etc.

- 103 of the 159 of the patients did not sign the informed consents? Do the authors have an explanation for this drop-out?

Thank you very much for the clarifying question. We cannot help but agree that, unfortunately, many patients refused to participate in the study. Most of them cited the unwillingness to leave home and travel to the study site as the reason.

Discussion: CREST terminology is used in Estonia, perhaps the authors should denote that this terminology is not anymore being used and rather use the term limited cutaneous SSc subset in this manuscript to avoid confusion.

Thank you for this important suggestion. It has been taken into account, and the addition has been implemented in the discussion section.

- More than 50% of patients were exposed to corticosteroids. It would be interesting to know how many patients had a renal crisis?

Thank you very much for this important question. Upon reviewing the information available to us, only one patient was also histologically diagnosed with a renal crisis.

- References: no year of publication is seen in the reference style

Thank you. The corrections have been implemented.

- Discussion 42-47: I do not understand the sentence: "In contrast, if the focus is on the time of diagnosis, we will not so much assess the characteristics of the disease as the availability of specialists in different countries."

Thank you very much for this very useful question. The sentence is indeed ambiguous, so we changed the sentence structure, which is also reflected in the manuscript, with the hope of a more unambiguous context.

“By contrast, if the focus remains on the time of diagnosis, we may mistakenly assess not the characteristics of the disease but the availability of specialists in different countries.”

- In the discussion it should be stated why the authors did not report on the incidence rate.

Thank you very much for this helpful suggestion, and an explanation has been included in the discussion section.

“The study did not analyse incidence data for systemic sclerosis. The main reason for this choice is missing data in hospital databases, and relying only on the medical history from patients can lead to very misleading data.”

- Typo's introduction line 38 : a spectrum of diseases that consists of…
